# An adaptive adjacency matrix-based graph convolutional recurrent network for air quality prediction

**DOI:** 10.1038/s41598-024-55060-2

**Published:** 2024-02-22

**Authors:** Quanchao Chen, Ruyan Ding, Xinyue Mo, Huan Li, Linxuan Xie, Jiayu Yang

**Affiliations:** School of Cyberspace Security/School of Cryptology, Hainan University, Haikou, China

**Keywords:** Air quality prediction, Spatio-temporal correlation, Graph convolutional network, Bayesian optimization, Deep learning, Environmental social sciences, Information technology

## Abstract

In recent years, air pollution has become increasingly serious and poses a great threat to human health. Timely and accurate air quality prediction is crucial for air pollution early warning and control. Although data-driven air quality prediction methods are promising, there are still challenges in studying spatial–temporal correlations of air pollutants to design effective predictors. To address this issue, a novel model called adaptive adjacency matrix-based graph convolutional recurrent network (AAMGCRN) is proposed in this study. The model inputs Point of Interest (POI) data and meteorological data into a fully connected neural network to learn the weights of the adjacency matrix thereby constructing the self-ringing adjacency matrix and passes the pollutant data with this matrix as input to the Graph Convolutional Network (GCN) unit. Then, the GCN unit is embedded into LSTM units to learn spatio-temporal dependencies. Furthermore, temporal features are extracted using Long Short-Term Memory network (LSTM). Finally, the outputs of these two components are merged and air quality predictions are generated through a hidden layer. To evaluate the performance of the model, we conducted multi-step predictions for the hourly concentration of PM_2.5_, PM_10_ and O_3_ at Fangshan, Tiantan and Dongsi monitoring stations in Beijing. The experimental results show that our method achieves better predicted effects compared with other baseline models based on deep learning. In general, we designed a novel air quality prediction method and effectively addressed the shortcomings of existing studies in learning the spatio-temporal correlations of air pollutants. This method can provide more accurate air quality predictions and is expected to provide support for public health protection and government environmental decision-making.

## Introduction

With the acceleration of industrialization and urbanization, the global air pollution problem is becoming increasingly severe, especially for adverse effects from air pollutants such as particulate matter (PM) and ozone (O_3_), which has aroused widespread concern^1^. Air pollution causes about 7 million premature deaths each year, with over 99% of the population living in areas where air pollutant concentrations exceed specified air quality standard, causing countless health hazards and economic losses^2^. PM and O_3_ are major pollutants of air pollution, seriously affecting the cardiovascular and respiratory systems and even other organs of human. In view of the harm of air pollution and severe air pollution situation, many organizations have set up air quality monitoring stations and published real-time air quality data for air quality monitoring and assessment^3^. However, for air pollution, it is not enough to merely monitor air quality, the prediction of pollutant concentrations is also crucial. Accurate air quality predictions enable the proactive identification of high-risk periods and regions, providing advance notice. This enables the public to promptly take necessary measures to protect themselves from potential health hazards. In addition, effective prediction can provide a scientific basis for government in air pollution prevention, formulating environmental policies and urban planning.

In recent years, data-driven air quality forecast methods have been widely used due to their advantages of high prediction accuracy, strong real-time performance, convenient implementation, and adaptive flexibility^4^. In general, the main methods can be divided into three types: statistical learning, traditional machine learning and deep learning methods^5–7^.

Statistical learning methods rely on sophisticated probabilistic modeling, predicting air quality through rigorous mathematical derivations and reasoning. A typical example is the Autoregressive Integrated Moving Average Model (ARIMA), and it has been widely applied due to its powerful processing capabilities for time-series data. Gourav et al.^8^ utilized the ARIMA model to perform modeling and forecasting on time series data. The performance of the model was evaluated by comparing the predicted results from the ARIMA model with the actual observations, and satisfactory and reliable outcomes were obtained. But the parameter selection of the ARIMA model usually requires specific expertise, and the prediction accuracy could vary significantly when faced with long-term forecast. Yu et al.^9^ introduced the Multivariable linear regression model (MLR) that takes into account multiple air pollution factors, allowing for a comprehensive understanding and prediction of air pollution conditions. In general, statistical learning is mainly based on linear assumptions and requires data to be stationary or transformable into stationary series through differencing. For some nonlinear or non-stationary data, its ability may be limited.

Traditional machine learning methods have demonstrated their effectiveness in data analysis and feature engineering, allowing for efficient data processing, transformation, and extraction of relevant features for modeling purposes. there are some classic and important models including the Decision Tree, the Support Vector Regression (SVR), the artificial neural network (ANN) and gradient-boosting^10^. For example, Althuwaynee et al.^11^ utilized multiple decision tree algorithms for classification analysis of air pollution index, and the best classifier was recommended based on performance comparison. Dun et al.^12^ integrated SVR into the grey multivariable regression model to predict air pollutant concentrations. By using the absolute percentage error (APE) to determine the weights of SVR, they aimed to enhance the accuracy and predictive capability of the model. As for the artificial neural network, Li et al.^13^ combined backpropagation neural networks with the Particle Swarm Optimization (PSO) to achieve rapid prediction and optimization of air quality with a limited number of computational fluid dynamics runs. These models can automatically learn nonlinear relationships and hidden features in data, thus discovering the inherent connections in data. However, the structure of these methods is relatively simple, limiting their data mining capabilities, and the prediction performance still needs to be further improved.

Compared with traditional machine learning, deep learning methods learn data representation by building a multi-layer neural network, and each layer is composed of a large number of neurons and learnable parameters. These methods have exhibited excellent performance in addressing challenges such as large sample sizes, high dimensionality, and nonlinearity^14^. In the task of prediction, deep learning can learn more hidden attributes of spatial and temporal data. In terms of spatial features, the convolutional neural network (CNN) is often used to extract spatial correlations from regional multi-site data. The graph convolutional neural network (GCN)^15^ aggregates features through the adjacency information of nodes, fully leveraging the spatial structure characteristics among air monitoring stations for air quality forecast. The graph attention network (GAT) proposed by Velickovic et al.^16^ uses an adaptive attention mechanism to dynamically learn the importance between different nodes, effectively capturing spatial correlations. As for temporal feature, the long short-term memory network (LSTM) could capture long-term dependencies and extract temporal correlations from data. The Transformer model^17^ and the BERT model^18^ use self-attention mechanisms to effectively handle long-term dependencies and complex spatio-temporal relationships.

Deep learning methods can extract more hidden attributes from time, space and other data, and could handle the complex spatio-temporal dependencies in air quality indices^19,20^. Therefore, researchers have further studied deep learning methods. In using deep learning methods to process spatio-temporal characteristics in air quality data, Yan et al.^21^ utilized the Convolutional Neural Network with Long Short-Term Memory (CNN-LSTM) model. This combination of two deep learning models allows the CNN-LSTM model to effectively integrate spatial and temporal characteristics when handling air quality prediction tasks. But this model has certain deficiencies, such as unsuitable for the grid structure of air quality data and ignoring complex correlations between nodes. To address the issues of the CNN-LSTM, Liu et al.^22^ adopted the Graph Convolutional Long Short-Term Memory (GC-LSTM) model, which combines graph convolution and long short-term memory networks. This model made significant progress in the comprehensive processing of space and time. However, for sparse graph structure data, GC-LSTM still has the problem of incomplete information transmission. Ma et al.^23^ proposed the Spatial–Temporal Graph Convolutional Network (ST-GCN) model, which combines graph convolutional network and temporal convolutional network to better handle graph structure data, especially data where spatial and temporal characteristics are interdependent. But ST-GCN still has limitations in handling data with dynamic spatial dependencies. And Liang et al.^24^ proposed the Geographical Multi-Attention Network (Geo-MAN) model, which combines geographic information and multiple attention mechanisms to achieve spatio-temporal interaction, thus better handling spatio-temporal dependencies. However, Geo-MAN cannot be directly applied to topological structures, which limits its scope of use. Furthermore, Huang et al.^25^ designed the Spatio-attention embedded recurrent neural network (SpAttRNN) model, which embeds graph attention cells into recurrent neural networks to handle dynamic spatial correlations.

Indeed, the prediction of air quality involves complex temporal and spatial dependencies. For instance, the concentration of air pollutants can be affected by geographical location and meteorological conditions^26^. Existing deep learning methods still have limitations in dealing with complex spatio-temporal dependencies, such as restrictions on data structure usage and inadequate performance in spatial dependencies and sparse data. In recent years, Graph Convolutional Network (GCN) have demonstrated excellent performance in dealing with non-grid data^27^. By performing convolution operations on the graph, GCN propagates and aggregates node features, thus better capturing dependencies among nodes and spatial patterns. The problem of air quality prediction is related to the geographical locations of various monitoring stations, which aligns with the structure of GCN. GCN also has the adaptability to sparse graph data and can handle graph structures with incompletely connected nodes. GCN could thus represent a promising avenue for advancing the field of air quality prediction. With their graph-based structure, they can leverage the geospatial data inherent to the problem, capturing the complex dependencies that exist between different monitoring sites and over time. Moreover, the capability of GCN to handle sparse and irregularly structured data makes them particularly suitable for this task where the data from different monitoring stations are uneven and sparse. Hence, it's worth exploring how GCN could be utilized and further improved for the task of air quality prediction.

Although current deep learning methods in the field of air quality prediction such as CNN-LSTM and GC-LSTM have made progress, there are still problems such as poor handling of grid structures, and incomplete information transfer of sparse graph structures with dynamic spatial dependency limitations. Therefore, this study designed two functional components to extract spatio-temporal dependencies and time features for air quality prediction. The first component proposed a novel adaptive spatial embedding recursive neural network based on GCN to capture the correlations of spatio-temporal data. It utilized a fully connected neural network to construct an adaptive self-loop adjacency matrix, which was combined with gas data as input to the GCN layer. This effectively overcomes the shortcomings of existing models by capturing node relationships and spatial patterns. Subsequently, the GCN was coupled with LSTM units to learn spatio-temporal dependencies. The second component utilized Long Short-Term Memory network (LSTM) to extract temporal features. Finally, the outputs of these two components were combined and passed through a hidden layer to generate air quality predictions. This provides a more comprehensive spatio-temporal dependent treatment scheme for air quality prediction, which promises superior performance in complex environments.

Our main contributions could be summarized as follows:

- A spatial feature extraction cell based on a self-loop normalized adjacency matrix is designed. Different from traditional graph attention units, the proposed adaptive self-loop normalized adjacency matrix combined with Graph Convolutional Neural Network can make the improved GCN be not limited by prior knowledge and better at extracting spatial features.
- A spatio-temporal encoder is proposed, which embeds the spatial feature extraction cell into the LSTM time feature learner and uses a coupled neural architecture to learn dynamic spatio-temporal dependencies.
- Bayesian automatic parameter search is employed for hyperparameter optimization, including hidden layer dimension and learning rate. Bayesian automatic parameter search models the probability distribution of hyperparameters and automatically adjusts the search strategy, effectively improving the efficiency and effectiveness of hyperparameter estimation.

## Results and discussion

### Hyperparameter adjustment

The choice of historical time window size (time lag) and correlated monitoring stations has a certain influence on the prediction result. And the selection of the correlation coefficient threshold determines the number of monitoring stations taken into account in the prediction task. Therefore, predictions were conducted for PM_2.5_, PM_10_, and O_3_ at Fangshan, Tiantan and Dongsi monitoring stations to compare the RMSE of the predicted results with different time lags and correlation coefficient thresholds on the validation set. Taking Fangshan monitoring station as an example, Table 1 clearly shows the lowest RMSE (normalized value) and optimal hyperparameters for the prediction of hourly pollutant concentration, namely correlation thresholds (time lags) are 0.75 (72), 0.7 (72) and 0.75 (120) for PM_2.5_, PM_10_ and O_3_ respectively.

**Table 1:** RMSE of predicted results with different hyperparameters.

Pollutant	Correlation threshold	Time lag					
		24	48	72	96	120	144
PM_2.5_	0.7	0.00711	0.00710	0.00743	0.00722	0.05002	0.01405
	0.75	0.04749	0.04646	0.00700	0.04844	0.00706	0.00948
	0.8	0.06249	0.00954	0.00932	0.00975	0.00888	0.01369
PM_10_	0.7	0.00871	0.00897	0.00855	0.00884	0.01710	0.01790
	0.75	0.00884	0.00881	0.00922	0.00900	0.01361	0.01402
	0.8	0.01741	0.01084	0.01104	0.01195	0.01460	0.01862
O_3_	0.7	0.01687	0.02389	0.07028	0.06101	0.07809	0.01690
	0.75	0.05828	0.05542	0.10183	0.06369	0.01572	0.02537
	0.8	0.12418	0.01838	0.02919	0.06860	0.02184	0.02682

Same processes were performed for the Tiantan and Dongsi monitoring stations. The optimal hyperparameters for Tiantan are 0.7 (120), 0.7 (24) and 0.75 (72) for PM_2.5_, PM_10_ and O_3_ respectively. And 0.7 (48), 0.7 (24) and 0.7 (72) are optimal hyperparameters for PM_2.5_, PM_10_ and O_3_ respectively in Dongsi.

Furthermore, Bayesian optimization algorithm is applied to automatically optimize three other hyperparameters of model, namely learning rate, input dimensions of FC Layer 2 and FC Layer 3 in Feature Merge module. The results of the Bayesian optimization are shown in Table 2, which are used in next prediction experiments.

**Table 2:** Hyperparameter selection by Bayesian Optimization.

Monitoring station	Pollutant	Learning Rate	Dimensions of the FC layer 2	Dimensions of the FC layer 3
Fangshan	PM_2.5_	0.00256	110	220
	PM_10_	0.00265	220	124
	O_3_	0.00254	116	162
Tiantan	PM_2.5_	0.00630	209	127
	PM_10_	0.00734	160	39
	O_3_	0.00953	192	126
Dongsi	PM_2.5_	0.0085	245	123
	PM_10_	0.00111	226	69
	O_3_	0.00998	221	76

### Prediction performance and comparison

In this study, the performance of the proposed AAMGCRN model is validated by case studies of multi-step prediction (7 steps) for the hourly concentration of PM_2.5_, PM_10_ and O_3_, and meanwhile AAMGCRN is compared with other classical benchmark models and advanced deep learning-based integrated models under same experimental conditions. Experiments were conducted at the Fangshan, Tiantan and Dongsi monitoring stations to verify the robustness and generalizability of the AAMGCRN. Detailed information of comparison models are as follows:

1. *LSTM* This is a recurrent neural network model that captures long-term dependencies in time series data by learning and maintaining memory units.
2. *Seq2Seq* The sequence-to-sequence network has two layers of recurrent neural networks, one RNN layer maps the input sequence to a feature vector, and the other layer decodes the feature vector into the target sequence for output.
3. *CNN-LSTM*^20^ This method uses convolutional neural network to extract spatial features from time series data, then feeds the extracted feature sequence into a Long Short-Term Memory network for time modeling and prediction.
4. *GC-LSTM*^21^ Use the GCN layer to learn the topological features of the nodes, and treat them as the input of the LSTM layer for modeling time dependencies.
5. *SpAttRNN*^25^ Through the embedded recurrent neural network, the model can capture long-term dependencies in time series data and extract important spatial features through a spatial attention mechanism.

As shown in Table 3, at Fangshan monitoring station, the results show that in the 7-steps prediction of PM_2.5_, the proposed AAMGCRN model ranks first among all deep learning models, and its RMSE, MAE and R^2^ are 19.11, 13.58 and 0.84 respectively. Compared with the second model LSTM, the RMSE and MAE decreased by 8.6% and 7.1% respectively, while R^2^ increased by 3.7%. Compared with the worst-performing model CNN-LSTM, the RMSE and MAE decreased by 25.8% and 26.2% respectively, and R^2^ increased by 16.7%. Compared with the SpAttRNN with complex structure and strategy, the RMSE and MAE decreased by 15.5% and 12.8% respectively, and R^2^ increased by 7.7%. For PM_10_, AAMGCRN also has the optimal performance, with RMSE, MAE, and R^2^ 23.73, 18.07 and 0.71 respectively. Compared with the second-ranked LSTM, RMSE and MAE decreased by 1.8% and 0.3% respectively, and R^2^ increased by 1.4%. Compared with the worst-performing CNN-LSTM, RMSE and MAE decreased by 19.4% and 21.4% respectively, and R^2^ increased by 29.1%. In predicting O_3_, AAMGCRN has similar prediction effects, with RMSE, MAE, and R^2^ 14.74, 11.07, and 0.54 respectively. Compared with the second-ranked Seq2Seq, RMSE, and MAE decreased by 0.5% and 1.0% respectively, and R^2^ increased by 14.9%. Compared with the advanced SpAttRNN, RMSE and MAE decreased by 10.1% and 11.3%, and R^2^ increased by 25.6%. In general, the proposed AAMGCRN model outperforms the benchmark models in prediction accuracy on all evaluation metrics.

**Table 3:** Predictive results of six deep learning models.

	PM_2.5_			PM_10_			O_3_		
	RMSE	MAE	R^2^	RMSE	MAE	R^2^	RMSE	MAE	R^2^
Fangshan monitoring station									
LSTM	20.9	14.62	0.81	24.16	18.13	0.70	15.71	12.33	0.4
Seq2Seq	21.03	14.71	0.81	26.44	20.11	0.64	14.81	11.18	0.47
CNN-LSTM	25.74	18.4	0.72	29.44	23.00	0.55	17.06	13.43	0.3
GC-LSTM	22.06	15.95	0.79	24.56	18.97	0.69	17.82	14.48	0.33
SpAttRNN	22.62	15.58	0.78	24.41	19.24	0.69	16.4	12.48	0.43
AAMGCRN	19.11	13.58	0.84	23.73	18.07	0.71	14.74	11.07	0.54
Tiantan monitoring station									
LSTM	20.64	13.46	0.81	23.87	17.58	0.66	16.91	12.92	0.35
Seq2Seq	21.49	13.35	0.79	22.35	16.52	0.71	15.67	11.71	0.44
CNN-LSTM	24.21	17.80	0.74	24.18	18.63	0.66	16.03	12.71	0.41
GC- LSTM	20.53	14.00	0.81	18.90	13.93	0.71	17.01	12.65	0.46
SpAttRNN	20.77	14.13	0.81	19.37	14.66	0.69	17.14	12.68	0.46
AAMGCRN	19.10	12.67	0.84	18.80	13.87	0.71	15.44	11.47	0.56
Dongsi monitoring station									
LSTM	21.88	14.03	0.81	20.59	15.11	0.78	17.67	12.80	0.27
Seq2Seq	21.27	13.94	0.82	21.5	15.96	0.76	16.77	12.49	0.34
CNN-LSTM	24.46	17.38	0.77	24.65	19.11	0.69	20.83	18.57	-0.01
GC-LSTM	20.61	14.04	0.83	19.25	14.39	0.77	16.64	14.45	0.44
SpAttRNN	20.19	13.40	0.84	18.45	13.77	0.79	16.93	13.27	0.42
AAMGCRN	18.21	12.04	0.87	17.75	13.02	0.80	14.39	11.05	0.58

In order to verify the generalization ability of AAMGCRN, air pollutant concentration data from the Tiantan and Dongsi monitoring stations were also used for case studies. From the prediction results, we can see that the proposed model has better prediction results, which is similar to Fangshan monitoring station. The AAMGCRN model is superior to all benchmark models for all pollutants based on evaluation metrics. For example, the RMSE, MAE, and R^2^ of the AAMGCRN are 18.80, 13.87 and 0.71 for PM_10_ at Tiantan monitoring station. Compared with CNN-LSTM, the RMSE and MAE decreased by 22.2% and 25.6% respectively, and the R^2^ increased by 7.6%. It could be concluded that AAMGCRN has high accuracy and applicability and can be well adapted to various pollutants in different environments.

The AAMGCRN is competitive in all performance metrics compared with other models, which can be further analyzed. In the prediction of air pollutant concentration, the LSTM and seq2seq model couldn’t effectively process the spatio-temporal correlation features, which is not enough to ensure satisfactory results. CNN-LSTM, although dealing with the features of spatial structure with the help of convolutional neural networks, is not fit for the pollutant data with grid architecture, which leads to poor results. Despite the efforts of the GC-LSTM and SpattRNN in capturing the spatio-temporal dependence features in the prediction task, they do not really learn the geographic location relationships among air quality monitoring stations to simulate actual dispersion process of air pollutants. It should be noted that the simple models outperform the complex models in the experiments, which means the complex architecture and strategies of the model don’t equate to excellent performance if it fails to properly handle the mixed attributes of data. In contrast, the first component designed in this study employs an adaptive spatially embedded recurrent neural network based on GCN, which constructs an adaptive self-looping neighbor matrix via a fully connected neural network, which is combined with the gas data as an input passed to the GCN layer, and subsequently combined with an LSTM unit to robustly learn the spatio-temporal dependence. The second component then utilizes the Long Short-Term Memory (LSTM) network to extract temporal features in a targeted manner. Ultimately, the outputs of these two components are combined and passed through the hidden layer to generate comprehensive and accurate air quality predictions.

This strategy of combining GCN and LSTM makes the model more robust in capturing spatio-temporal dependencies, while the component focusing on temporal features further improves the comprehensive performance of the prediction, making it more suitable for complex air quality prediction tasks. Meanwhile, this study considered the effects of the historical time window size and the selection of the correlation monitoring stations on the prediction results, and the prediction experiments were conducted at three monitoring stations. By comparing the RMSE of the prediction results with different historical time window sizes and correlation coefficient thresholds in the validation set, the effects of these two factors on the model performance were explored in depth. This approach provides a strong reference for further model optimization. Meanwhile, the strategy proposed in this study uses Bayesian automatic parameter search for hyper-parameter optimization, effectively improving the efficiency and effectiveness of hyperparameter estimation. All these advantages are verified by the experimental results.

**Figure 1:**
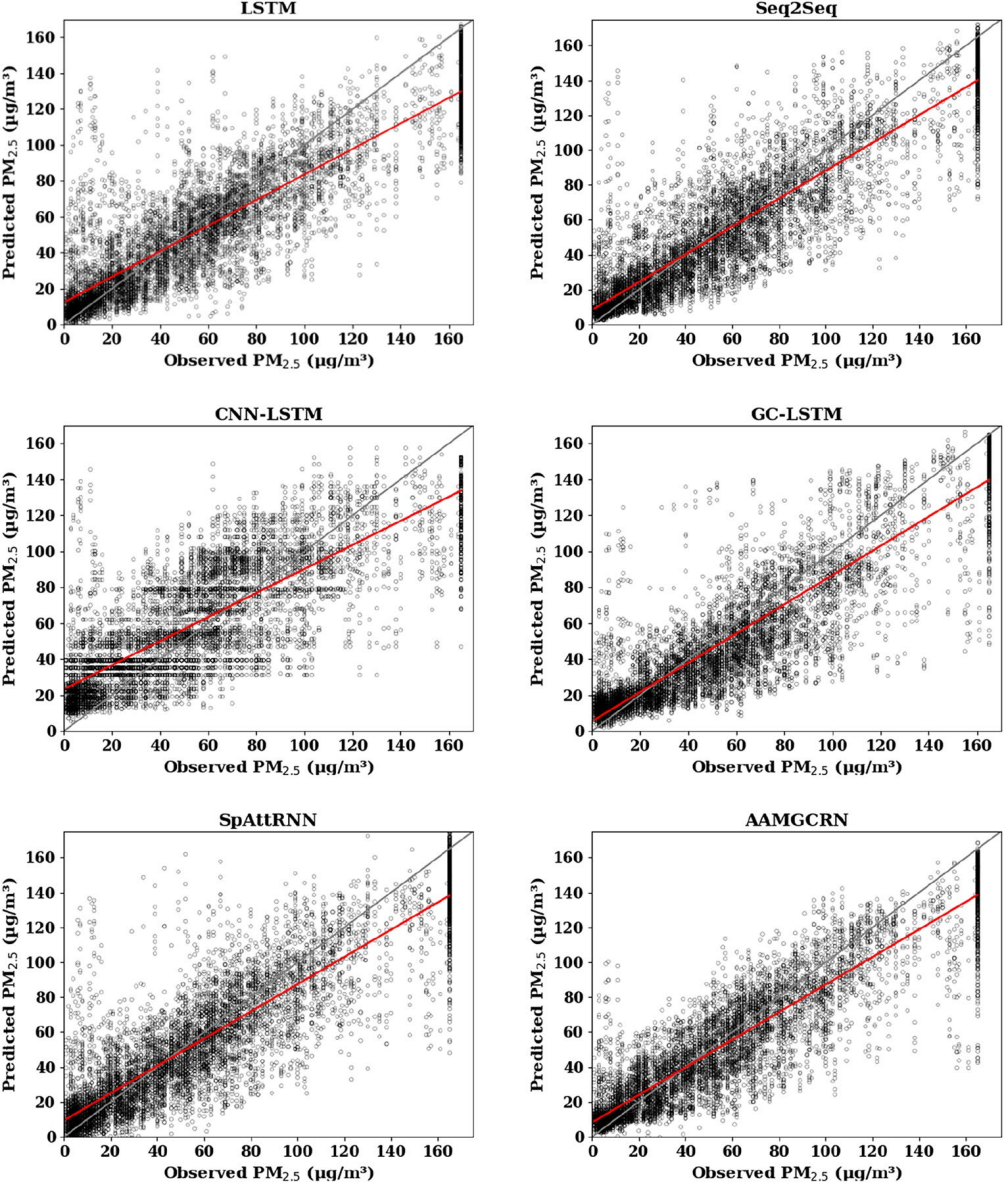
Scatter plot of predicted PM_2.5_ results at Fangshan monitoring station.

**Figure 2:**
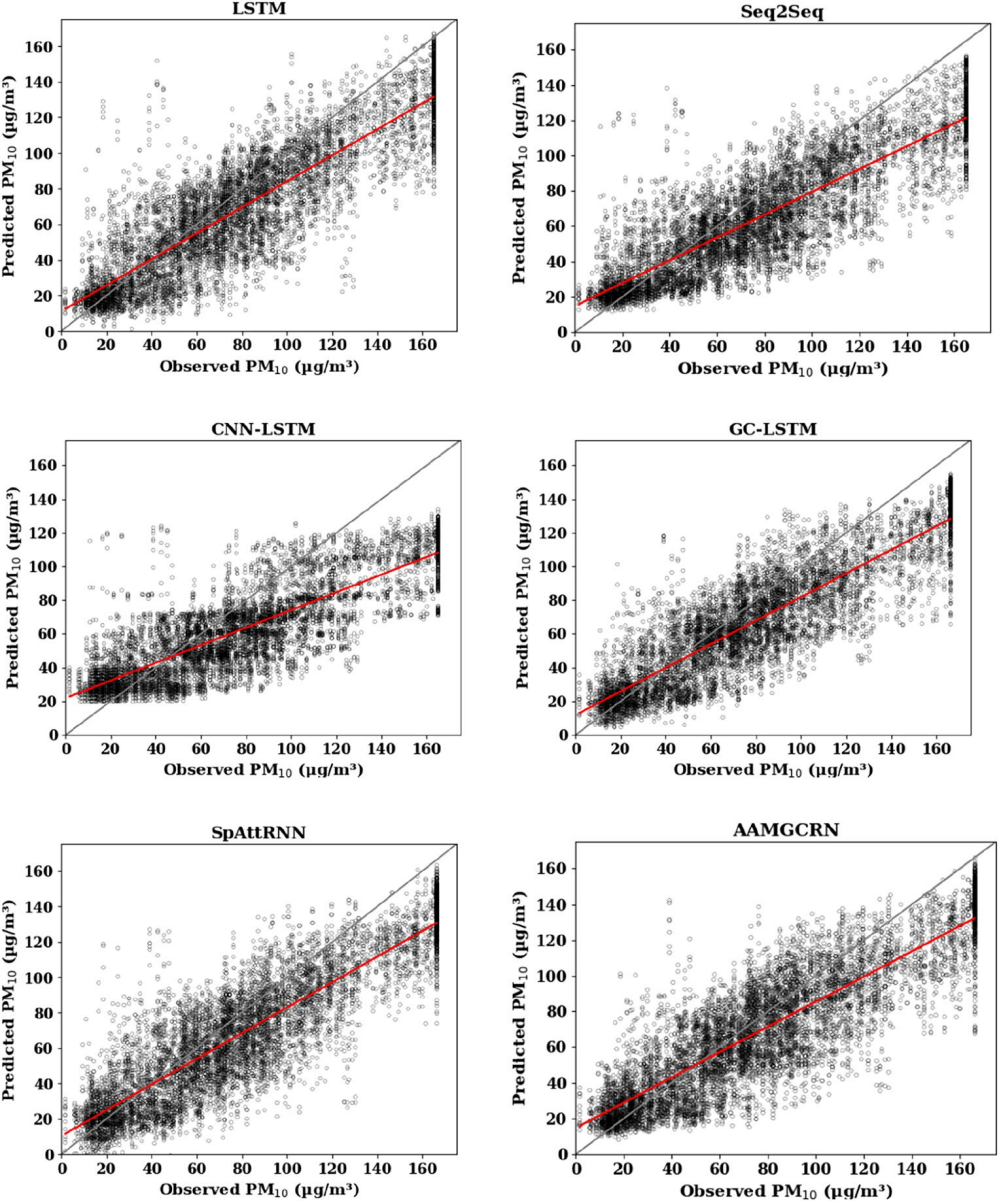
Scatter plot of predicted PM_10_ results at Fangshan monitoring station.

**Figure 3:**
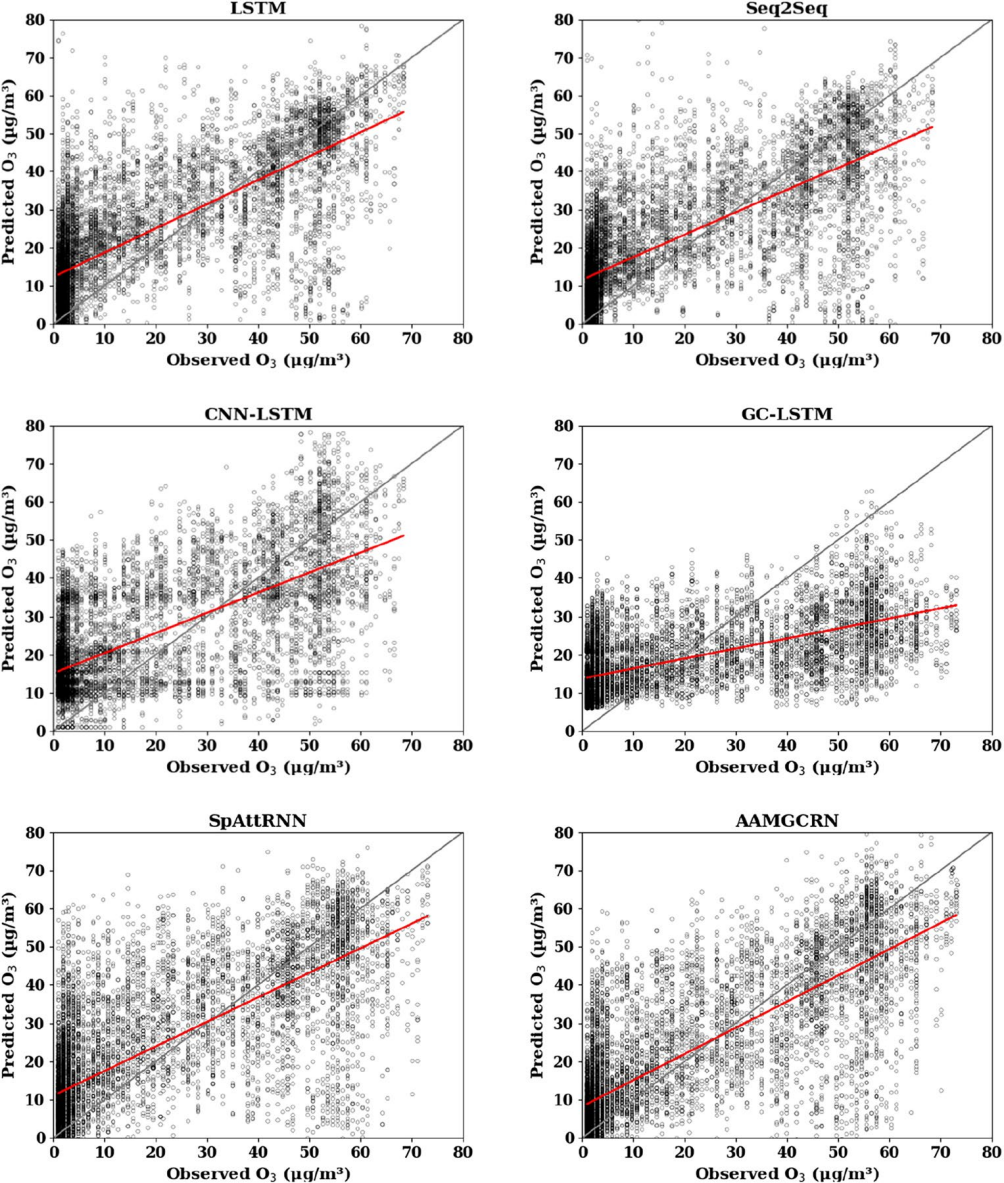
Scatter plot of predicted O_3_ results at Fangshan monitoring station.

In order to clearly exhibit the difference between predicted value and observed value, scatterplot is adopted to visualize predictive results of all prediction models. Take Fangshan as an example, Figs. 1, 2 and 3 show the comparisons of prediction and observation results of the six models in the prediction of PM_2.5_, PM_10_, and O_3_ respectively.

The solid gray line in the figure is the 1:1 standard line, and the red line is the regression line between the predicted values and observed values. The closer the red line is to the gray line, the closer the predicted value is to the true value, and the better the prediction effect is. It can be seen that the red line of the AAMGCRN model is closer to the gray line than the other models at Fangshan monitoring station. Its R^2^ are 0.84, 0.71 and 0.54 for PM_2.5_, PM_10_ and O_3_ respectively, which reflects the effectiveness and applicability of AAMGCRN in predicting pollutant concentration based on the effective extraction of spatial and temporal information.

### Sensitivity analysis

To understand the impact of each component of AAMGCRN, this study compares the predictive results of complete AAMGCRN model with its core modules as independent predictors, namely Spatio-temporal self-learning module and Temporal self-learning module. The Spatio-temporal self-learning module employs an adaptive spatially embedded recurrent neural network based on GCN to construct an adaptive self-looped adjacency matrix via a fully connected neural network and incorporates gas data as the input to the GCN layer. This module focuses on capturing the correlation of spatio-temporal data to deal with the complex associations between nodes and realizes the spatio-temporal dependence learning. The temporal self-learning module employs the Long Short-Term Memory (LSTM) network, which focuses on extracting the time-series features of meteorological data. This module realizes targeted extraction of time-related features in meteorological data through LSTM structure for processing information in time-series data.

It can be seen from Table 4 that the prediction performance of AAMGCRN benefitting from the combination of the Spatio-temporal self-learning module and Temporal self-learning module is generally better than that of them alone, which proves that the two modules are mutually beneficial. It can be concluded that considering only Spatio-temporal feature or temporal feature is not sufficient, and an effective combination of both features is necessary to obtain better prediction results.

**Table 4:** Sensitivity analysis results of AAMGCRN.

Monitoring station	Predictor	PM_2.5_			PM_10_			O_3_		
		RMSE	MAE	R^2^	RMSE	MAE	R^2^	RMSE	MAE	R^2^
Fangshan	Spatio-temporal self-learning module	19.96	13.88	0.83	23.97	18.61	0.71	17.88	14.01	0.32
	Temporal self-learning module	20.17	14.61	0.83	25.62	19.59	0.66	15.43	11.04	0.50
	AAMGCRN	19.11	13.58	0.84	23.73	18.07	0.71	14.74	11.07	0.54
Tiantan	Spatio-temporal self-learning module	22.18	15.66	0.78	19.00	14.33	0.70	20.22	16.24	0.24
	Temporal self-learning module	20.73	13.78	0.81	19.88	14.77	0.68	15.68	11.28	0.55
	AAMGCRN	19.10	12.67	0.84	18.80	13.87	0.71	15.44	11.47	0.56
Dongsi	Spatio-temporal self-learning module	24.20	17.06	0.77	19.21	14.59	0.77	17.53	13.60	0.37
	Temporal self-learning module	22.08	14.86	0.81	19.59	14.41	0.76	14.56	11.49	0.56
	AAMGCRN	18.21	12.04	0.87	17.75	13.02	0.80	14.39	11.05	0.58

In addition, this study compares the effects of Bayesian optimization and empirical selection on hyperparameters on the prediction performance. The hyperparameters of empirical selection are from previous research^28^. The hyperparameters obtained by Bayesian optimization and empirical selection are shown in Table 5, and the comparison results are shown in Table 6. Compared with the empirical selection of hyperparameters, the model optimized by Bayesian optimization shows an advantage in prediction. For example, at Fangshan monitoring station, the RMSE and MAE for the PM_2.5_ prediction were reduced by 10.4% and 12.7%, respectively, and the R^2^ was improved by 3.7%. For O_3_, the RMSE and MAE decreased by 4.0% and 1.7%, respectively, and the R^2^ improved by 7.8%. For the PM_10_ prediction task, RMSE and MAE decreased by 10.9% and 12.2%, respectively, and R^2^ improved by 9.9%. The performance improvement can also be clearly seen in the experimental results from the Tiantan and Dongsi monitoring stations. It proves that Bayesian optimization has improved the performance of the model. The optimization of hyperparameters using Bayesian method can not only improve the predictive ability of the model, but also save computational resources. Compared with empirical selection and trial-and-error methods, Bayesian optimization has a solid mathematical theoretical foundation and good interpretability.

**Table 5:** Bayesian optimization and Empirical selection.

Monitoring station	Hyperparameter	Priori experience			Bayesian optimization		
		PM_2.5_	PM_10_	O_3_	PM_2.5_	PM_10_	O_3_
Fangshan	Learning rate	0.01	0.01	0.01	0.00256	0.00265	0.00254
	Dimensions of the FC layer 2	10	10	10	110	220	116
	Dimensions of the FC layer 3	128	128	128	220	124	162
Tiantan	Learning rate	0.01	0.01	0.01	0.00630	0.00734	0.00953
	Dimensions of the FC layer 2	10	10	10	209	160	192
	Dimensions of the FC layer 3	128	128	128	127	39	126
Dongsi	Learning rate	0.01	0.01	0.01	0.00850	0.00111	0.00998
	Dimensions of the FC layer 2	10	10	10	245	226	221
	Dimensions of the FC layer 3	128	128	128	123	69	76

**Table 6:** Predictive results of empirical selection and Bayesian optimization.

Monitoring station	Method	PM_2.5_			PM_10_			O_3_		
		RMSE	MAE	R^2^	RMSE	MAE	R^2^	RMSE	MAE	R^2^
Fangshan	Empirical selection	21.32	15.55	0.81	26.63	20.59	0.64	15.36	11.26	0.50
	Bayesian optimization	19.11	13.58	0.84	23.73	18.07	0.71	14.74	11.07	0.54
Tiantan	Empirical selection	19.79	13.18	0.83	19.55	14.51	0.69	16.38	12.17	0.50
	Bayesian optimization	19.10	12.67	0.84	18.80	13.87	0.71	15.44	11.47	0.56
Dongsi	Empirical selection	21.87	14.98	0.81	20.94	15.81	0.73	14.46	10.82	0.57
	Bayesian optimization	18.21	12.04	0.87	17.75	13.02	0.80	14.39	11.05	0.58

## Discussion

This study proposes a model called Adaptive Adjacency Matrix-Based Graph Convolutional Recurrent Network (AAMGCRN) for air quality prediction. To investigate the complex spatio-temporal interactions at different stations, this approach constructs an adaptive self-loop adjacency matrix using a fully connected neural network and combines pollutant data as input to the GCN layer. The GCN is then combined with LSTM units to learn spatio-temporal dependencies. Additionally, AAMGCRN extracts long sequence time dependencies from meteorological and air quality data using LSTM. Together, these components effectively learn the complex relationships between air pollutants and the surrounding environment.

Compared with existing deep learning models, the spatial feature extraction cell based on a self-loop normalized adjacency matrix is proposed, which means the improved GCN is not limited by prior knowledge and can better extract spatial features. Meanwhile, the spatio-temporal encoder proposed in this study will embed the spatial feature extraction cell into the temporal feature learner LSTM, which can better learn the dynamic spatio-temporal dependencies. Furthermore, the AAMGCN employs Bayesian automatic parameter search for hyperparameter optimization, effectively improving the efficiency and effectiveness of hyperparameter estimation.

And then the AAMGCRN model is tested through case studies with integrated evaluations and sensitivity analysis using hourly pollutant concentration and meteorological element data as well as POI data in Beijing. The experimental results show that AAMGCRN outperforms the benchmark models in predicting PM_2.5_, PM_10_ and O_3_. Take Fangshan monitoring station as an example. For the 7-steps prediction of PM_2.5_ hourly concentration, the RMSE, MAE, and R^2^ were 19.11, 13.58, and 0.85, respectively. For PM_10_, the RMSE, MAE, and R^2^ of AAMGCRN were 23.73, 18.07, and 0.71, respectively. For O_3_, the RMSE, MAE, and R^2^ were 14.74, 11.07 and 0.54. The experimental results at the Tiantan and Dongsi monitoring stations were also optimal compared to the other baseline models.

In the future, we will continue to optimize the network structure and parameter settings to enhance the extraction of spatio-temporal dependency features of data and improve the prediction performance of model. Additionally, the proposed approach and strategy could also be applied to other scenarios of spatio-temporal sequence mining, such as wind speed and traffic flow prediction.

## Limitations of the study

The complexity of model leads to a large number of parameters and time-consuming tuning process. This is because the use of Bayesian hyperparameter tuning increases the overall training time. In Bayesian hyperparameter tuning, multiple model training and evaluation are required in order to construct the agent model, which can increase the overall number of training sessions. Furthermore, Bayesian tuning involves serialized parameter attempts because the choice of the next parameter combination depends on the performance of the previous combination, which complicates parallel training. In future work, we will explore adaptive learning methods to reduce computing time while improving prediction accuracy.

In this study, we assume that the POI data remain constant throughout the prediction process. The change of POI may influence predicted results, even though this is unlikely to happen during a short period. This point will be considered more comprehensively in future.

## Data and methods

### Data

#### Data description

The data used in this study include three categories: pollutant concentration, meteorological factor and Point of Interest (POI) information. Hourly data from 2018.1 to 2018.12 for six conventional air pollutants (PM_2.5_, PM_10_, NO_2_, CO, O_3_, SO_2_) are collected from environmental monitor stations in Beijing (https://github.com/DHA-AI4VN2022/MAML/tree/main/data/Beijing). Corresponding meteorological data contain humidity, surface temperature, wind speed, precipitation, barometric pressure, and optical radiation. These meteorological factors influence the transport and transformation processes of pollutants. Wind speed, temperature, humidity and, weather phenomena influence dispersion conditions. For example, wind can transport pollutants horizontally, while wind speed affects the speed and distance at which pollutants are transported. Temperature and humidity affect atmospheric stability and vertical dispersion, while precipitation is important for removing air pollutants. Barometric pressure is directly related to air density and affects the conditions for the dispersion of pollutants in the atmosphere. Optical radiation contributes to the generation or breakdown of air pollutants, such as ozone, by triggering photochemical reactions in the atmosphere. POI can well represent the static geospatial characteristics of the monitoring station, and the number of landmark buildings near monitoring station can be used as POI data. MapWorld API provides seven types of POI data in the Beijing area, namely cafe, amusement park, university, factory, school, shopping center and park. In general, there is a temporal pattern for air pollutant, and its properties are influenced by pollution source and meteorological condition. The data of pollutant concentration, meteorological factor and POI could provide much related information, which helps our model AAMGCRN explore the correlations among the predicted pollutant concentration and related features. In view of the prominent effects of PM_2.5_, PM_10_ and O_3_, they are treated as predicted pollutants for case studies. Furthermore, the 60%, 25% and 15% of the dataset are used as the training set, validation set and test set respectively.

**Figure 4:**
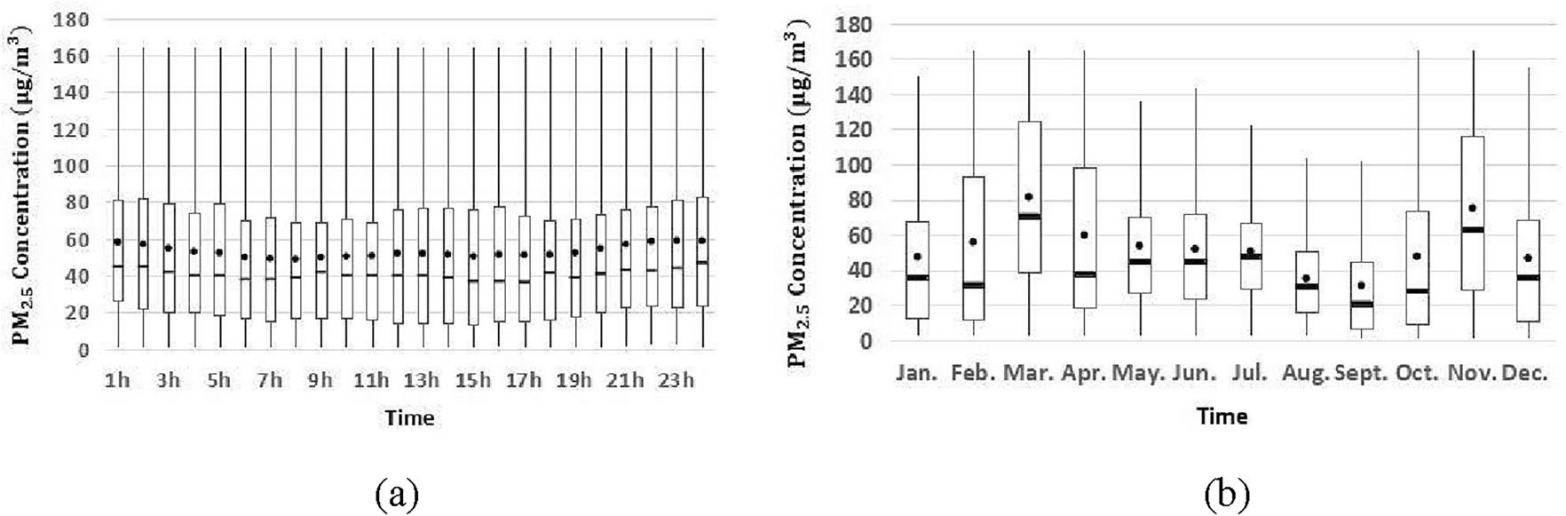
Hourly (**a**) and monthly (**b**) variation of PM_2.5_ concentration in 2018.

**Figure 5:**
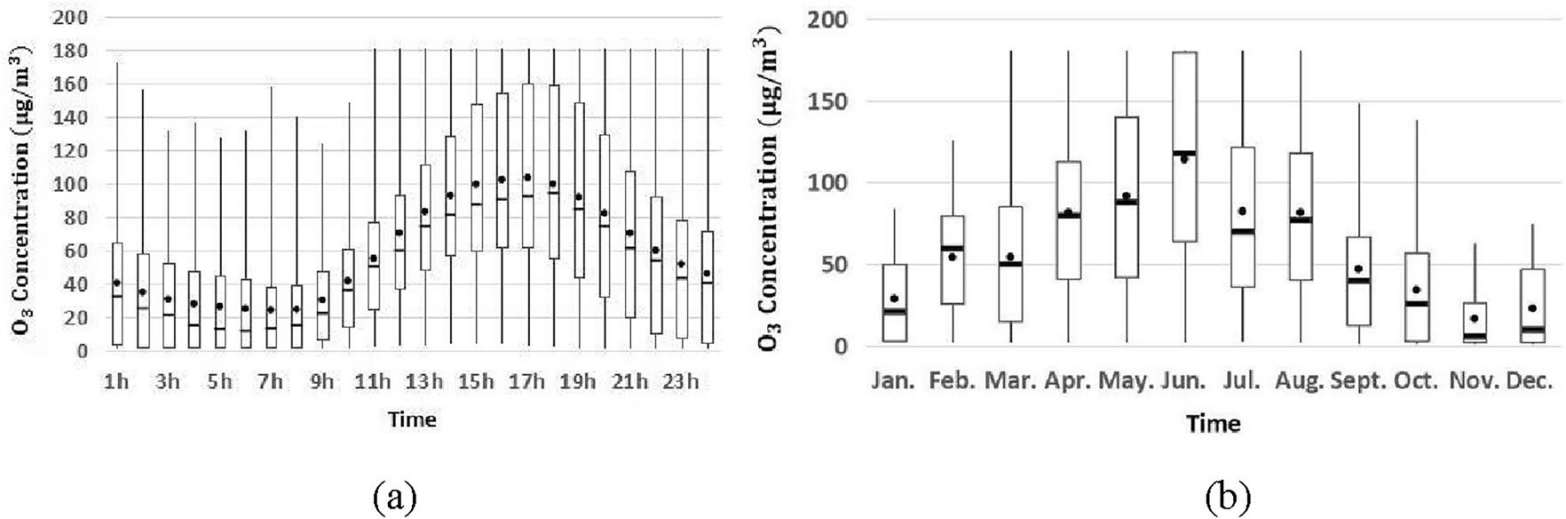
Hourly (**a**) and monthly (**b**) variation of O_3_ concentration in 2018.

**Figure 6:**
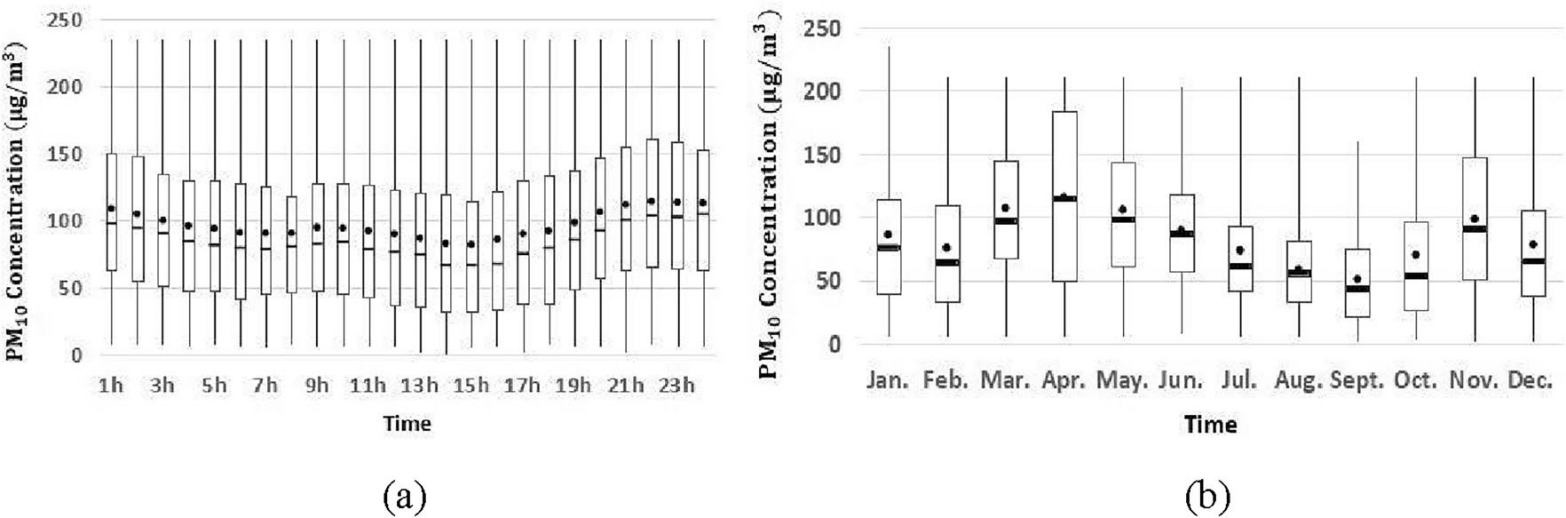
Hourly (**a**) and monthly (**b**) variation of PM_10_ concentration in 2018.

For the predicted pollutants at the target station, the time series data is visualized with the Fangshan monitoring station as an example (Figs. 4, 5 and 6). PM_2.5_ retains high concentration during one day, and there is an increase after the morning and evening rush hour. And it climbs significantly in autumn and winter, but the opposite phenomenon exists in spring and summer. There are more notable temporal patterns of O_3_ for hourly and monthly variation, and it reaches maximum value at 16 h and in June respectively. The hourly and monthly variations of PM_10_ show a clear temporal pattern, with its concentration showing a significant upward trend at night and a significant downward trend in the summer months. Similar temporal patterns occur at Tiantan and Dongsi monitoring stations too, and detailed statistical information of pollutant concentration is given in Table 7.

**Table 7:** Statistical features of predicted pollutant time series.

Monitoring station	Pollutant	Maximum	Minimum	Median	Mean	Standard deviation
Fangshan	PM_2.5_	165	1	41	53.22	44.66
	PM_10_	235	1	84	96.32	61.73
	O_3_	181	1	48	58.67	52.18
Tiantan	PM_2.5_	151	1	36	47.81	41.61
	PM_10_	183	2	66	75.84	47.04
	O_3_	172	1	48	55.83	48.17
Dongsi	PM_2.5_	164	1	38	51.37	44.94
	PM_10_	211	2	71	82.52	54.95
	O_3_	180	1	52	61.3	50.52

### Methods

#### Data preprocessing

Considering the influence of all monitoring stations on predicting the pollutant concentration at the target monitoring station may introduce redundant information. To mitigate this issue, data preprocessing was conducted, and monitoring stations with a strong correlation with the target monitoring station were identified through correlation analysis. This screening process helps to reduce the interference of irrelevant information.

Correlation analysis is a commonly used statistical analysis method to assess the degree of relationship or interconnection between variables. In this study, Pearson's correlation coefficient is used to measure the correlation between monitoring stations. The Pearson correlation coefficient^29^ was used to calculate the correlation between the concentration of a specific pollutant at the target monitoring station and the concentration of the same pollutant at other monitoring stations. A correlation coefficient closer to 1 indicates a higher correlation between the variables. The calculation formula is shown below.

1 $$r = \frac{{\mathop \sum \nolimits_{i = 1}^{n} \left( {X_{i} - \overline{X}} \right)\left( {Y_{i} - \overline{Y}} \right)}}{{\sqrt {\mathop \sum \nolimits_{i = 1}^{n} (X_{i} - \overline{X})^{2} } \sqrt {\mathop \sum \nolimits_{i = 1}^{n} (Y_{i} - \overline{Y})^{2} } }}$$

where r denotes the Pearson correlation coefficient, and $\overline{X}$ and $\overline{Y}$ denote the mean values of the variables $X$ and $Y$, respectively.

Furthermore, normalization method is applied to eliminate the impact of magnitude among multiple variables. Min–Max normalization is a commonly used method of normalizing data by linearly scaling the original data to a specified range [0, 1]. The formula is

2 $$x\_new = \frac{x - \min (x)}{{\max (x) - \min (x)}}$$

where $x$ denotes the original data, $x\_new$ denotes the normalized data, and $\min (x)$ and $\max (x)$ denote the minimum and maximum values of the original data, respectively.

### Evaluation metric

To objectively evaluate the prediction performance of model, three classical statistical indicators are adopted, namely Root Mean Square Error (RMSE), Mean Absolute Error (MAE) and Coefficient of Determination (R^2^). For RMSE and MAE, the smaller the value, the better the predictive performance. For R^2^, the closer the value is to 1, the better the predictive performance of the model. Their formulas are as follows:

Root Mean Square Error (RMSE):

3 $$RMSE = \sqrt {\frac{1}{N}\mathop \sum \limits_{i = 1}^{N} (F_{i} - O_{i} )^{2} }$$

Mean Absolute Error (MAE):

4 $${\text{MAE}} = \frac{1}{{\text{N}}}\mathop \sum \limits_{{{\text{i}} = 1}}^{{\text{N}}} \left| {{\text{F}}_{{\text{i}}} - {\text{O}}_{{\text{i}}} } \right|$$

Coefficient of Determination (R^2^):

5 $$R^{2} = 1 - \frac{{\mathop \sum \nolimits_{i = 1}^{N} (F_{i} - O_{i} )^{2} }}{{\mathop \sum \nolimits_{i = 1}^{N} (\overline{O} - O_{i} )^{2} }}$$

where N is the number of samples; $F_{i}$ and $O_{i}$ represent the actual and predicted values of the $i$th sample; $\overline{F}$ and $\overline{O}$ are the average of the actual and predicted values.

The RMSE is also used for the loss function, which is combined with Adam (Adaptive Moment Estimation) optimizer for optimization. The Adam optimizer is an adaptive optimization algorithm that can adjust the learning rate based on historical gradient information. It normalizes the update of parameters, making each parameter update of a similar magnitude, thereby improving the training effect.

### Model overview

#### Problem formulation

In order to tackle the problem of air quality prediction, a specific target monitoring station was selected as the subject of study. The concentration of a specific air pollutant for the next $\tau$ time steps is predicted by utilizing the space station network information and historical data provided by monitoring sites spanning $T$ time steps. The calculation formula is as follows:

6 $$\left\{ {X^{{t_{0} + 1}} ,X^{{t_{0} + 2}} , \ldots ,X^{{t_{0} + \tau }} } \right\} = f\left( {G;\left\{ {X^{{t_{0} - T + 1}} ,X^{{t_{0} - T + 2}} , \ldots ,X^{{t_{0} }} } \right\}} \right)$$

where $X^{{t_{0} + 1}} , \ldots ,X^{{t_{0} + \tau }}$ represent the predicted data, $X^{{t_{0} - T + 1}} , \ldots ,X^{{t_{0} }}$ represent the historical data, $G$ denotes the space site network data, and $f$ is the mapping function.

#### Feature explanation

It has been shown that pollutant concentration has dynamic spatial correlation and dynamic temporal correlation, and these properties will be further considered in modeling. Dynamic spatial correlation is reflected in the fact that the dispersion of pollutants in space can be affected by meteorological factors. For example, the transport and dispersion of pollutants may occur in response to changes of wind direction and speed, thus mutually affecting the air quality in different areas. Meanwhile, different pollution sources such as neighboring industrial zones could lead to more complex correlations. Therefore, it’s necessary to take complex dynamic spatial correlations into account in the prediction task. Dynamic temporal correlation refers to that pollutant concentration has periodic variations. For example, apparent pollutant concentration variations occur at different times of the day, and certain seasons may be more prone to pollutant accumulation or dispersion. Such cyclical variations can be attributed to factors such as people's living patterns and travel habits. Therefore, it is essential to consider the effect of historical time on the predicted time. For addressing the air pollution prediction task, it is essential to consider the dynamic spatial correlation and dynamic temporal correlation simultaneously. This approach enables more accurate predictions of future air quality.

### Model specification

#### Adaptive spatio-temporal self-learning module

(1) GCN unit based on adjacency matrix.

The POI data and meteorological data are inputted into the fully connected layer for learning the adjacency weight. This weight is then incorporated into the adjacency matrix. Subsequently, the pollutant concentration data is preprocessed and used as the input (X) for the Graph Convolutional Network (GCN), which is combined with the adjacency matrix to jointly obtain the spatially encoded output.

Graph convolutional network can effectively fuse node features and topology to extract graph data features. Therefore, in this study a spatio-temporal self-learning layer is built based on graph convolutional network. For the GCN unit, firstly, N nodes are given, and each node has its own features. Assume that these node features form an N*D-dimensional matrix X, and the relationship between each node forms an N*N-dimensional matrix A, which is known as the adjacency matrix. Here X and A are the inputs to the GCN. As a neural network layer, the GCN propagates from layer to layer in the following way:

7 $$H = \sigma \left( {\tilde{D}^{\frac{1}{2}} \tilde{A}\tilde{D}^{\frac{1}{2}} XW} \right)$$

where $\tilde{A} = A + I$, $I$ is the unit matrix, $\tilde{D}$ is the degree matrix of $\tilde{A}$, $\tilde{D} = \mathop \sum \limits_{j} \widetilde{{A_{IJ} }}$, and $H$ is the feature of each layer, $X$ is the input to the input layer. $\sigma$ is the ELU activation function as a nonlinear activation function.

However, the classical GCN still has some drawbacks. all elements in ${\tilde{\text{A}}}$ = A + I are set manually before training and do not change during training. If the values of the adjacency matrix were simply set to 0 or 1, this would mean that each different upstream node would have the same effect on the downstream node, which is not conducive to learning the interactions of the two monitoring stations and is not practical.

To address this issue, a trainable adaptive adjacency matrix is proposed in order to learn distinct weights between two monitoring stations. This allows the GCN to adjust the parameters automatically during the training process. The specific details of this approach are as follows.

8 $$Adapt_{A} = \sigma \left( {W_{Adapt} X_{spatial} } \right) \circ A$$

where $\circ$ denotes the Hadamard product, $\sigma$ is the activation function of the nonlinear model, here this study use ReLU^30^ as the activation function of the adaptive adjacency matrix. $W_{Adapt}$ is an $N*N$ matrix, where $N$ is the number of monitoring station nodes. Its shape is the same as the adjacency matrix $A$. $\left( {w_{i} ,w_{j} } \right)$ denotes the influence weight of the $i^{th}$ monitoring station on the $j^{th}$ monitoring station. $X_{spatial}$ is the input vector, including POI spatial information as well as meteorological data. ${\text{A}}$ is the inverse of the Euclidean distance of the two locations, which denotes the initialized adjacency matrix.

9 $$f_{gcn} \left( {\tilde{X},Adapt_{A} } \right) = \sigma \left( {\tilde{D}^{\frac{1}{2}} Adapt_{A} \tilde{D}^{\frac{1}{2}} XW} \right)$$

As a result, by training the effects between different nodes in the adjacency matrix, they can be made to vary independently while capturing dynamic spatial correlation features. The final GCN unit equation is shown in Eq. (9).

(2) Spatio-temporal LSTM adjacency layer.

The data in the adjacency matrix is fed into the Long Short-Term Memory Network Embedded with Graph Convolutional Network (LSTMEGCN) as one input. Simultaneously, the processed pollution concentration data is directly inputted into the LSTMEGCN as another input.

**Figure 7:**
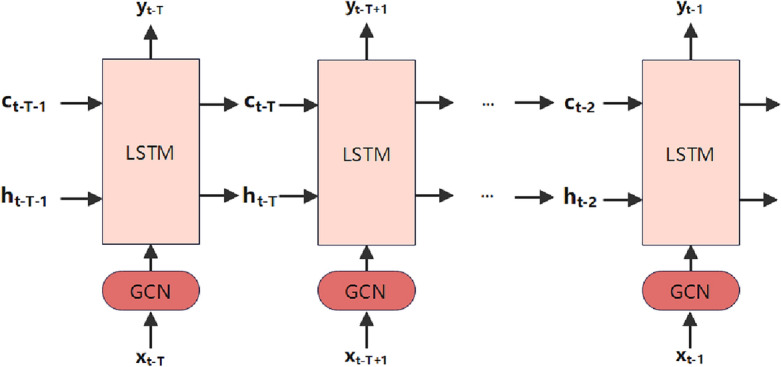
Details of Adaptive Spatio-Temporal Self-learning Module.

After considering the dynamic spatial correlation, the module continues to focus on the dynamic temporal dependence. Compared with traditional RNN, LSTM is often used to process and model sequential data with stronger memory and long-term dependency modeling capabilities. The key point is that it introduces a gating mechanism to solve the problems such as gradient vanishing and gradient explosion in traditional RNN by controlling the flow of information and memory updating. The basic unit of LSTM consists of a cellular state c and three gating units including an input gate i, a forgetting gate f, and an output gate o. To enhance the learning of spatio-temporal dependence features, the GCN unit is integrated into the temporal LSTM self-adaptation layer, creating a novel spatio-temporal dependent self-learning module. This module is illustrated in Fig. 7. It is specifically represented as

10 $$f_{\left( t \right)} = \sigma_{g} \left( {W_{f} f_{gcn} \left( {\hat{X},Adapt_{A} } \right) + U_{f} Z_{t - 1}^{st} + b_{f} } \right)$$

11 $$i_{\left( t \right)} = \sigma_{g} \left( {W_{i} f_{gcn} \left( {\hat{X},Adapt_{A} } \right) + U_{i} Z_{t - 1}^{st} + b_{i} } \right)$$

12 $$o_{\left( t \right)} = \sigma_{g} \left( {W_{o} f_{gcn} \left( {\hat{X},Adapt_{A} } \right) + U_{o} Z_{t - 1}^{st} + b_{o} } \right)$$

13 $$c_{\left( t \right)} = f_{t} \circ c_{t - 1} + i_{\left( t \right)} \circ \sigma_{h} \left( {W_{c} f_{gcn} \left( {\hat{X},Adapt_{A} } \right) + U_{c} Z_{t - 1}^{st} + b_{c} } \right)$$

14 $$Z_{\left( t \right)}^{st} = o_{\left( t \right)} \circ \sigma_{h} \left( {c_{\left( t \right)} } \right)$$

where $W$ and $U$ denote the weight matrices of the control gating units, $b$ is the bias vector, and $o$ is denotes the Hadamard product. $\sigma_{g}$ is the activation function sigmoid^31^, and $\sigma_{h}$ is the hyperbolic tangent function.

#### Temporal self-learning module

To extract additional time-dependent features, a self-time learning LSTM layer is introduced to capture temporal patterns of the target monitoring station. This layer is responsible for learning temporal features such as pollution concentration changes and meteorological information. The input data is meteorological data and air quality data. The time self-learning is specifically denoted as

15 $$f_{\left( t \right)} = \sigma_{g} \left( {W_{f} X_{t} + U_{f} Z_{t - 1}^{self} + b_{f} } \right)$$

16 $$i_{\left( t \right)} = \sigma_{g} \left( {W_{i} X_{t} + U_{i} Z_{t - 1}^{self} + b_{i} } \right)$$

17 $$o_{\left( t \right)} = \sigma_{g} \left( {W_{o} X_{t} + U_{o} Z_{t - 1}^{self} + b_{o} } \right)$$

18 $$c_{\left( t \right)} = f_{t} \circ c_{t - 1} + i_{\left( t \right)} \circ \sigma_{h} \left( {W_{c} X_{t} + U_{c} Z_{t - 1}^{self} + b_{c} } \right)$$

19 $$Z_{\left( t \right)}^{self} = o_{\left( t \right)} \circ \sigma_{h} \left( {c_{\left( t \right)} } \right)$$

where $W$ and $U$ denote the weight matrices of the control gating units, $b$ is the bias vector, and $o$ is denotes the Hadamard product.

#### Feature merge module

The outputs of the Spatio-Temporal Self-learning module and Temporal Self-learning module are concatenated and spliced to generate the final feature vector. This feature vector is converted into the final prediction result through the fully connected network layer. This is represented as

20 $$\left[ {\widehat{{y_{T + 1} }}, \ldots ,\widehat{{y_{T + T} }}} \right] = W_{{z_{T}^{{\left( {st + self} \right)}} }} + b$$

where $W$ is the weight matrix of the hidden layer, $b$ is the bias vector of the hidden layer, and $T^{\prime }$ is the prediction length of the multi-step prediction of air quality.

#### Bayesian automatic parameter tuning

There are some hyperparameters in neural network, such as learning rate, batch size, etc. The selection of hyperparameters has always been a key issue for deep learning models. In order to further improve the prediction accuracy of model, Bayesian optimization algorithm^32^ is applied to search for the optimal hyperparameters. Bayesian optimization can use the information of the searched points to guide the next search, improving the quality of the next search as well as the overall search speed. The goal of Bayesian optimization is to find the d-dimensional hyperparameter that minimizes the loss value in the hyperparameter space. The overall formula is as follows:

21 $$x^{*} = argmin\,\,loss\left( x \right)$$

where $x$ is the set of input hyperparameters and $x^{*}$ is the optimal combination of hyperparameters after Bayesian optimization. In the model, the hyperparameters that are optimized are the learning rate of the overall model and the dimension of the hidden layer. These hyperparameters are crucial factors for achieving optimal performance and are adjusted during the training process. The hidden layer dimensions include the output dimensions of the Temporal Self-learning Module and the output dimensions of the Feature Merge Module. $loss\left( x \right)$ expresses the model generalization metrics about the model hyperparameters, and here this study take the root mean square error as loss function.

22 $$loss\left( {x_{j} } \right) = \sqrt {\frac{1}{n}\mathop \sum \limits_{i = 1}^{n} (\widehat{{y_{I} }}\left( {x_{j} } \right) - y_{i} )^{2} }$$

where $x_{j}$ is the $j{\text{th}}$ hyperparameters combination, $y$ is the actual value, and $\widehat{{y_{I} }}\left( {x_{j} } \right)$ is the model output using the hyperparameters combination $x_{j}$. The $loss$ function is a black-box objective function with a high evaluation cost, and the goal of Bayesian optimization is to find the optimal hyperparameters combination at a smaller cost. The available evaluation data:

23 $$D_{1:p} = \left( {x_{i} ,loss\left( {x_{i} } \right)} \right)$$

where $i = 1,2, \ldots ,t$. $P$ represents the known data.

The probabilistic proxy model is set up to estimate the distribution of the objective function. The Gaussian process can refine the model by continuously adding information to the data based on the kernel function and the observations, all of which follow a normal distribution in form.

24 $$x^{*} = argmin\,\,loss\left( x \right)P\left( {loss} \right) = N\left( {loss;\mu ,k} \right)$$

25 $$P(loss|D) = N(loss;\mu \_(loss|D),k\_(loss|D))$$

where $N$ is the Gaussian distribution^33^, $k$ is the variance, and $\mu$ is the mean.

The acquisition function α(∙)is defined to measure the impact that the observation points have on the fitted model. The acquisition function consists of the obtained posterior distribution and performs the next observation based on the point with the highest impact.

26 $$x_{t + 1} = argmax\,\,a_{t} \left( {x;D} \right)$$

Based on the new observations added to the acquisition function, the root mean square error loss value is continuously optimized to find the set of hyperparameters corresponding to its minimum time. The process is looped over and over until the number of iterations or the maximum allowed time is reached and stopped.

#### The architecture of AAMGCRN

**Figure 8:**
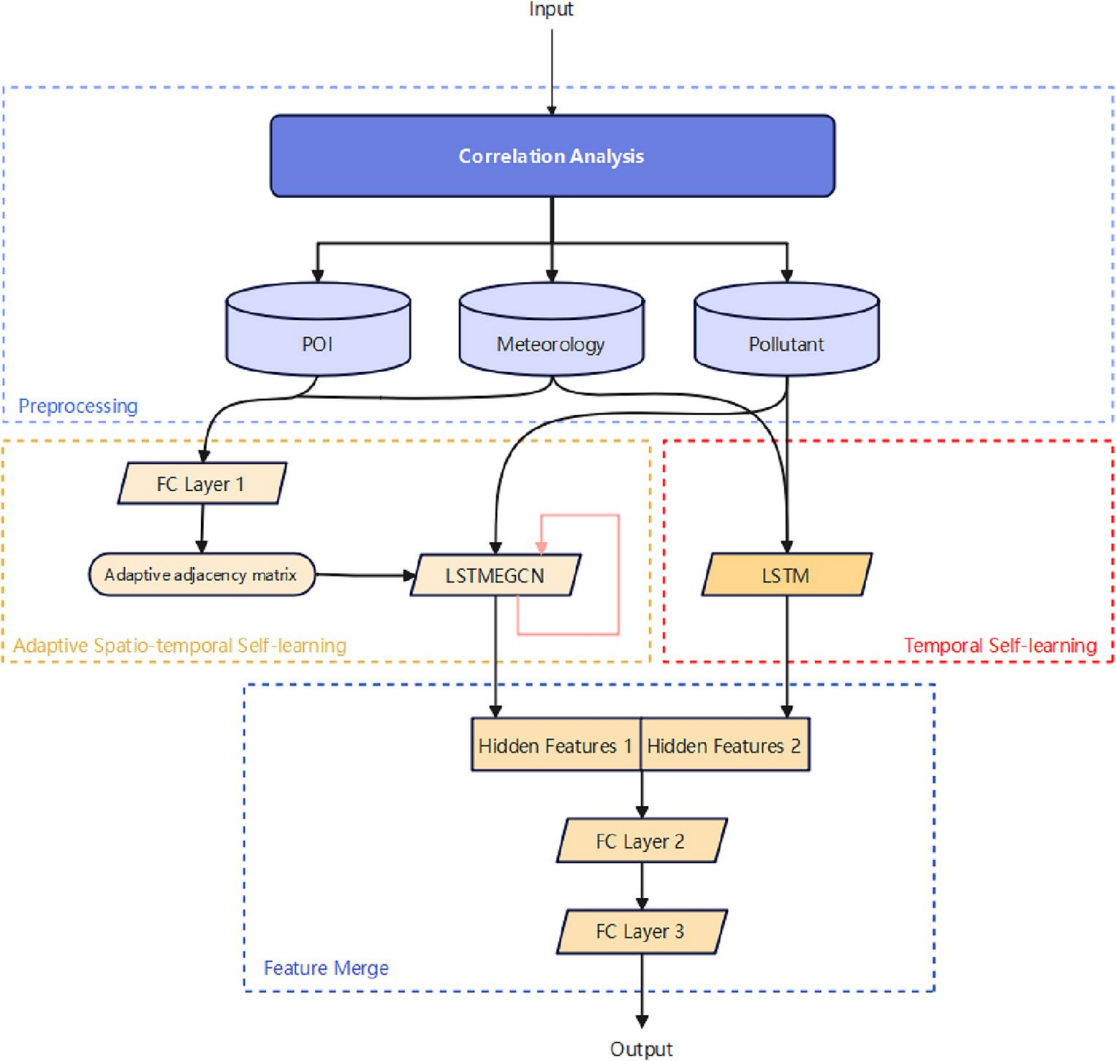
The architecture of AAMGCRN.

The proposed network is outlined in Fig. 8, and the overall process includes data preprocessing and the AAMGCRN model. The AAMGCRN model mainly consists of three parts: (1) Adaptive Spatio-Temporal Self-learning module, which learns the spatio-temporal dependencies from static data (POI) and dynamic data (meteorological data and air quality data); (2) Temporal Self-learning module, which extracts long sequence time dependencies from dynamic data (meteorological data and air quality data); (3) Feature Merge module, which combines the outputs of the two modules and generates the prediction results through a hidden layer

## Data Availability

All data are stored in Github and have been made publicly available. The address is: https://github.com/A-train-Bestman/AAMGCRN_data. Code will be provided by corresponding author by moxinyue@hainanu.edu.cn when requested.
